# Modelling the effect of beliefs about asthma medication and treatment intrusiveness on adherence and preference for once-daily vs. twice-daily medication

**DOI:** 10.1038/s41533-017-0061-7

**Published:** 2017-11-14

**Authors:** Sarah Chapman, Peter Dale, Henrik Svedsater, Gillian Stynes, Nicola Vyas, David Price, Rob Horne

**Affiliations:** 1 University of Bath, Bath, UK; 2 GSK, Brentford, UK; 3 Healthcare Research Worldwide, Wallingford Oxon, UK; 4 Observational and Pragmatic Research Institute, Singapore, Singapore; 5 University College London, London, UK; 6 Present Address: Gillian Stynes, Bristol-Myers Squibb, Uxbridge, UK; 7 Present Address: Peter Dale, HEOR Solutions Ltd, Marlow, UK

## Abstract

People with asthma who do not adhere to their maintenance medication may experience poorer asthma control and need more healthcare support than those who adhere. People (*N* = 1010) aged 18–55 years with self-reported asthma, taking one or more asthma maintenance medication(s), from five European countries, participated in a survey using validated scales (Medication Adherence Report Scale [MARS], Asthma Control Test™ [ACT], Beliefs about Medicine Questionnaire [BMQ] and the Asthma Treatment Intrusiveness Questionnaire [ATIQ]). We performed a post hoc evaluation of adherence to maintenance medication, asthma control, beliefs about medication, preferences for once-daily vs. twice-daily asthma maintenance medication and treatment intrusiveness, using structural equation modelling to investigate the relationships between these factors. Most participants reported potential problems with asthma control (ACT < 19: 76.8% [*n* = 776]), low adherence (median MARS = 3.40) and preferred once-daily medication (73.5% [*n* = 742/1010]). Non-adherence was associated with worse asthma control (*r* = 0.262 [*P* < 0.001]) and a expressed preference for once-daily medication over a "twice daily medication that works slightly better" (test statistic [*T*] = 2.970 [*P* = 0.003]). Participants reporting non-adherence/preferring once-daily medication had negative beliefs about their treatment (BMQ necessity-concerns differential: *r* = 0.437 [*P* < 0.001]/*T* = 6.886 [*P* < 0.001]) and found medication intrusive (ATIQ: *r* = −0.422 [*P* < 0.001]/*T* = 2.689[*P* = 0.007]). Structural equation modelling showed complex relationships between variables, including: (1) high concerns about treatment associated with increased perceived treatment intrusiveness and reduced adherence, which influenced asthma control; (2) high concerns about treatment and healthcare seeking behaviour, which were predictive of preferring twice-daily asthma medication. Concerns about medication and perceived treatment intrusiveness were predictive of poor adherence, and were associated with preference for once-daily asthma medication. Confirm the utility of the PAPA model and NCF in explaining nonadherence linked to poor asthma control.

## Asthma: Examining beliefs around maintenance medication

Encouraging adherence to maintenance medication for asthma requires a dual approach that considers patient perceptions as well as practicalities. While doctors may prescribe patients with appropriate medication to control asthma, many patients do not adhere to their maintenance regime. The reasons for this are complex, revolving around patient beliefs about long-term medication. Robert Horne at University College London, UK, and co-workers modelled data collected from interviews with 1010 adult patients from five European countries. Over a quarter of patients reported non-adherence and this was linked to concerns about ICS and perceived treatment intensiveness. Patients' beliefs about ICS and the concept of medication 'intrusiveness' were key factors in non-adherence. Understanding individual patient goals, eliciting patient concerns and addressing practical barriers should help encourage adherence.

## Introduction

Asthma is a common chronic condition, with an estimated global prevalence of 4.3–4.5%, with higher rates (5.3%) reported in urbanised regions, such as in Europe.^1^ Approximately 1 in 250 deaths globally are attributed to asthma,^2^ and the condition places a significant burden on patients, their families, healthcare systems and the economy, including morbidity, absenteeism and presenteeism, hospitalisation, reduced quality of life and costs.^3,4^

Despite a wide range of treatment options, many people continue to experience uncontrolled asthma, increasing the risk of morbidity, mortality and poor quality of life.^5^ While treatments do not effectively control symptoms in some people with asthma (who continue to experience exacerbations despite taking therapy as prescribed), many do not take their medication as prescribed (non-adherence)^6^ and consequently do not experience the full benefit from treatment. Non-adherence rates of 35–86% have been reported in people with asthma.^7,8^ Non-adherence includes several different types of behaviour, such as failure to initiate therapy, failure to take medication as instructed (implementation, which may result from having poor inhaler technique) and lack of persistence in continuing therapy as directed.^9,10^ Typically, non-adherence is examined as the latter two, which are possible to measure following treatment prescription.

It is important to identify people with asthma who are at risk of non-adherence, as non-adherence can be predictive of poor asthma control^11^ and adverse clinical outcomes, such as asthma exacerbations.^12–14^ Furthermore, understanding the reasons for non-adherence and identifying people who would prefer to switch to a once-daily treatment may aid physicians in prescribing the most appropriate treatment and thus maximise adherence.

Medication adherence is best understood as the product of two overlapping constructs: motivation and ability.^15^ Beliefs about treatment are important. Adherence is influenced by the patient’s evaluation of their treatment, particularly how they judge the necessity for the treatment relative to their concerns about the (actual and potential) adverse consequences of using it. The Necessity–Concerns Framework (NCF) includes these two aspects of patients’ perceptions that influence adherence,^16^ and the Beliefs about Medicines Questionnaire (BMQ) provides a brief, valid and reliable method for measuring these beliefs.^17^ Studies applying these approaches to maintenance treatment in asthma show that non-adherence is related to patients’ doubts about their personal need (necessity beliefs) and concerns.^16,18–22^ These beliefs may arise in part from how patients perceive their treatment experiences; for example, someone with asthma who is asymptomatic may not detect a change in symptoms from their asthma maintenance medication, potentially reinforcing a belief that they do not need to take it.^23^

The importance of patients’ beliefs about illness and treatment is recognised by the National Institute for Clinical Excellence medicines adherence guidelines (NICE),^24^ which takes a perceptions and practicalities approach (PAPA),^15,25^ advocating that adherence support should focus on perceptions (e.g., our beliefs about, and experiences of, the illness and treatment) as well as practicalities (e.g., capacity, resources and access to treatment) in order to follow the treatment recommendations. A key practical issue is the complexity of treatment regimens,^26^ and simplifying the regimen may be an example of an incentive to facilitate adherence.

Once-daily asthma maintenance medication regimens may reduce the burden of treatment, compared with more frequently required therapy, and thus improve adherence.^27–29^ However, the evidence for patient preference for, and adherence to, once-daily treatments remains limited. The PAPA may be useful to explore preference for once-daily treatments because identification of practical barriers to adherence for a patient’s current asthma treatment regimen may predict whether they wish to switch to a once-daily medication. In asthma, good control and low self-perceived controller medication need have been associated with once-daily medication preference.^30^

In this post hoc analysis of European survey data (Healthcare Research Worldwide, London, UK; ‘Once Daily Medication Taking Behaviour Research’ study), we assessed measures of adherence, perceptual barriers to asthma medication adherence (doubts about necessity and concerns about adverse effects) and practical barriers to treatment (the intrusiveness of asthma treatment), asthma control and healthcare seeking. Preferences for once-daily vs. twice-daily asthma maintenance medication were also investigated.

We hypothesised that non-adherence and preferences for switching to a ‘once-daily medication that works as well as my current medication’ or a ‘twice-daily medication that works slightly better than my current medication’ would be predicted by perceptual and practical factors associated with current treatment, and that non-adherence would predict healthcare-seeking behaviour. Complex pathways may exist between the variables that impact adherence and treatment frequency preferences; for example, people with a high perception of treatment need are likely to be more adherent and so less likely to seek healthcare as a result of uncontrolled asthma, or may engage more with their healthcare in general and so be more likely to seek healthcare. To explore the relationships between these variables, we used structural equation modelling.

## Results

### Participant characteristics

Overall, 1010 people with asthma were included in these analyses, and their demographic and clinical characteristics are presented in Table 1. The mean age was 36.6 years (standard deviation [SD] 10.2; range 18–55 years), and the median duration of asthma was 15 years (interquartile range [IQR] 7–23 years). Participants had experienced a median of two asthma attacks in their lifetime (IQR 0–3) and were taking a median of two asthma maintenance medications (IQR 1–3) (Table 1; full list in Supplementary Table S1). Asthma medication regimens corresponding to Step 3 of the global stepwise treatment framework^5^ were taken by 45.6% of participants, while 37.6% of participants were taking asthma medication regimens that corresponded to Step 2 of the framework, together accounting for 83.3% of all participants (Table 1).

**Table 1:** Participant characteristics and clinical factor

Participants		*N *= 1010	
Demographic characteristic		*n* (%)	
Country^a^	Germany	200 (19.8)	
	UK	204 (20.2)	
	Spain	201 (19.9)	
	France	206 (20.4)	
	Italy	199 (19.7)	
Gender	Male	499 (49.4)	
	Female	511 (50.6)	
Marital status	Married/cohabiting/living with partner	655 (64.9)	
	Single	284 (28.1)	
	Separated/divorced/widowed	71 (7.0)	
Area of residence	Urban	509 (50.4)	
	Rural	501 (49.6)	
Employment	Employed, full time	559 (55.3)	
	Employed, part time	98 (9.7)	
	Self-employed	97 (9.6)	
	Unemployed	81 (8.0)	
	Student	71 (7.0)	
	Home maker	59 (5.8)	
	Other	45 (4.5)	
Asthma characteristics	*Median (IQR)*		
	Age at asthma diagnosis	18.00 (10.00–30.00)	
	Years since asthma diagnosis	15.00 (7.00–23.00)	
	Number of lifetime asthma attacks	2.00 (0.00–3.00)	
Clinical characteristics	*n* (%)		
Smoking history	I’ve never smoked	451 (44.7)	
	I did smoke, but don’t smoke now	274 (27.1)	
	I only smoke at social occasions	65 (6.4)	
	I smoke less than 5 cigarettes a day on average	52 (5.1)	
	I smoke 5–15 cigarettes a day on average	107 (10.6)	
	I smoke over 15 cigarettes a day on average	61 (6.0)	
Severity^b^	Mild	139 (13.8)	
	Moderate	694 (68.7)	
	Severe	117 (11.6)	
	Not disclosed	60 (5.9)	
Medication regimen step^c^	Step 1	94 (9.3)	
	Step 2	380 (37.6)	
	Step 3	461 (45.6)	
	Step 4	32 (3.2)	
	Step 5	43 (4.3)	
Healthcare service use in relation to asthma in the prior year		*n (%)*	*Median (IQR)*
Consultations	GP	887 (87.8)	3 (1–5)
	Practice/community nurse	336 (33.3)	0 (0–1)
	Specialist/consultant	557 (55.1)	1 (0–2)
	Specialist nurse	207 (20.5)	0 (0–0)
	Dietician	150 (14.9)	0 (0–0)
	Other HCP	153 (15.1)	0 (0–0)
	All consultations	964 (95.4)	5 (2–10)
Emergencies	Emergency GP appointments	499 (49.4)	0 (0–1)
	Emergency service uses	361 (35.7)	0 (0–1)
	Overnight hospital stays following emergency care	204 (20.2)	0 (0–0)
	Taken to hospital by ambulance	166 (16.4)	0 (0–0)
	Sent to hospital by GP/specialist	243 (24.1)	0 (0–0)
	Hospitalisations	332 (32.9)	0 (0–1)
	Total days spent hospitalised	N/A	2 (1–5)

*GP*, general practitioner, *HCP* healthcare professional, *IQR* interquartile range, *N/A* not available

^a^ Country of recruitment

^b^ “How has your doctor described your asthma?”

^c^ Stepwise treatment framework (GINA, 2015, summary of medication at each regimen step: step 1, SABAs alone or in combination with allergy treatment; step 2, ICS alone or leukotriene modifiers or ICS in combination with SABAs or allergy-induced asthma treatment; step 3, LABAs in combination with ICS or theophylline/related compounds or ICS in combination with allergy-induced asthma treatment; step 4, ICS in combination with LABAs and allergy-induced asthma treatment; step 5, omalizumab)

The mean Asthma Control Test™ (ACT) score was 16.02 (SD 4.17). The majority of participants (76.8%, *n = *776) had ACT scores <19, indicating potential problems with asthma control, and 46.1% of participants (*n = *466) had scores <16, indicating poorly controlled or uncontrolled asthma. Only 1.8% of participants (*n* = 18) had ‘ideal’ asthma control, with an ACT score of 25.

There was a wide variety of healthcare-seeking frequencies among participants in the 12 months prior to the survey, with a median of three general practitioner consultations (range 0–60, sought by 87.8% of participants, *n = *887) and one specialist consultation (range 0–45, sought by 55.1% of participants, *n = *557); two-thirds of participants did not consult a community nurse (range 0–50, *n = *336) (Table 1).

### Participant scores and preferences

Participants’ adherence to their asthma maintenance medication was assessed using the Medication Adherence Report Scale (MARS). The median MARS score was 3.40 (IRQ 2.90–4.10). When MARS scores were dichotomised at approximately the lowest third of scores (<3), 72.4% (*n = *731) of participants had MARS scores indicating high adherence, and 27.6% (*n = *279) had scores indicating low adherence.

Perceived intrusiveness of participants’ maintenance asthma treatment was investigated using the Asthma Treatment Intrusiveness Questionnaire (ATIQ). Most participants had ATIQ scores indicative of low intrusion into their daily lives from their asthma maintenance medication, and the overall median ATIQ score was 26.00 (IQR 16.00–39.00; of a potential range 13.00–65.00) (Table 2).

**Table 2:** Participant scores for perceived treatment necessity, concerns about treatment and treatment intrusiveness

		Median (IQR)			
	*N *= 1010	BMQ necessity score^a^ (potential range: 1–5)	BMQ concerns score^a^ (potential range: 1–5)	BMQ necessity-concerns^b^ differential score	ATIQ total score^c^ (potential range: 13–65)
Gender	Female	3.60 (3.00–4.00)	2.44 (2.00–3.11)	0.80 (0.13–1.69)	23.00 (14.00–36.00)
	Male	3.40 (3.00–4.00)	2.89 (2.22–3.44)	0.38 (0.00–1.09)	33.00 (20.00–39.00)
Country^d^	Germany	3.60 (3.00–4.00)	2.56 (1.89–3.11)	0.69 (0.04–1.64)	25.50 (15.50–38.00)
	UK	3.60 (3.00–4.00)	2.39 (1.89–3.00)	0.91 (0.21–1.61)	19.50 (14.00–37.50)
	Spain	3.60 (3.00–4.00)	2.89 (2.44–3.44)	0.38 (0.00–0.98)	33.00 (19.00–40.00)
	France	3.80 (3.00–4.00)	2.56 (2.00–3.33)	0.66 (0.09–1.78)	25.00 (16.00–38.00)
	Italy	3.40 (2.00–4.00)	3.00 (2.22–3.56)	0.31 (0.00–1.07)	32.00 (19.00–40.00)
Marital status	Married/cohabiting/living with partner	3.60 (3.00–4.00)	2.67 (2.00–3.22)	0.49 (0.00–1.33)	26.00 (15.00–38.00)
	Other	3.40 (3.00–4.00)	2.78 (2.22–3.22)	0.62 (0.07–1.47)	29.00 (19.00–39.00)
Area of residence	Urban	3.60 (3.00–4.00)	2.78 (2.11–3.33)	0.44 (0.00–1.33)	28.00 (17.00–39.00)
	Rural	3.60 (3.00–4.00)	2.56 (2.00–3.11)	0.69 (0.09–1.60)	26.00 (15.00–38.00)
Employment	Full-time employment	3.60 (3.00–4.00)	2.78 (2.11–3.33)	0.49 (0.00–1.40)	30.00 (16.00–39.00)
	Other employment	3.60 (3.00–4.00)	2.56 (2.00–3.11)	0.64 (0.04–1.51)	25.00 (16.00–37.00)
Asthma severity^e^	Mild	3.20 (2.60–4.00)	2.44 (1.78–3.11)	0.47 (0.00–1.36)	19.00 (13.00–36.00)
	Moderate	3.60 (3.00–4.00)	2.89 (2.22–3.33)	0.51 (0.00–1.33)	30.00 (17.00–39.00)
	Severe	4.00 (3.40–4.40)	2.67 (2.00–3.33)	1.16 (0.18–2.11)	31.00 (18.00–39.00)
Medication regimen step^f^	Step 1	3.40 (3.00–4.00)	2.83 (2.22–3.33)	0.49 (0.00–1.16)	33.00 (20.00–40.00)
	Step 2	3.40 (3.00–3.80)	2.67 (2.00–3.11)	0.51 (0.00–1.27)	27.00 (16.00–39.00)
	Step 3	3.60 (3.00–4.00)	2.67 (2.00–3.11)	0.69 (0.09–1.67)	24.00 (15.00–37.00)
	Step 4	4.00 (3.40–4.20)	3.22 (2.06–4.00)	0.50 (−0.01–1.30)	36.50 (18.00–43.00)
	Step 5	3.60 (3.00–4.00)	3.11 (2.67–3.67)	0.11 (0.00–0.73)	38.00 (30.00–41.00)
Smoking	Current smoker	3.60 (3.00–4.00)	2.56 (2.00–3.11)	0.71 (0.07–1.64)	24.00 (14.00–38.00)
	Not current smoker	3.60 (3.00–4.00)	2.78 (2.11–3.33)	0.53 (0.00–1.36)	28.00 (17.00–39.00)
Current asthma medication	Overall score	3.60 (3.00–4.00)	2.67 (2.00–3.22)	0.58 (0.00–1.42)	26.00 (16.00–39.00)
Preference for treatment	Once-daily asthma medication	3.40 (3.00–4.00)	2.78 (2.11–3.44)	0.42 (0.00–1.24)	28.00 (16.00–39.00)
	Twice-daily asthma medication	3.80 (3.20–4.00)	2.44 (1.89–3.00)	1.01 (0.34–1.83)	24.00 (15.00–36.00)

*ATIQ* Asthma Treatment Intrusiveness Questionnaire, *BMQ* Beliefs about Medicines Questionnaire, *ICS* inhaled corticosteroid, *IQR* interquartile range, *LABA* long-acting beta_2_ agonist, *SABA* short-acting beta_2_ agonist

^a^ Rated on a 5-point Likert scale (from ‘strongly disagree’ = 1 to ‘strongly agree’ = 5)

^b^ BMQ Concerns score subtracted from BMQ Necessity score

^c^ Sum of 13 possible intrusions of asthma on participants’ daily lives, each rated on a 5-point Likert-type scale (from ‘low’ = 1 to ‘high’ = 5

^d^ Country of recruitment

^e^ “How has your doctor described your asthma?”

^f^ Stepwise treatment framework (GINA, 2015, summary of medication at each regimen step: step 1, SABAs alone or in combination with allergy treatment; step 2, ICS alone or leukotriene modifiers or ICS in combination with SABAs or allergy-induced asthma treatment; step 3, LABAs in combination with ICS or theophylline/related compounds or ICS in combination with allergy-induced asthma treatment; step 4, ICS in combination with LABAs and allergy-induced asthma treatment; step 5, omalizumab)

**Fig. 1:**
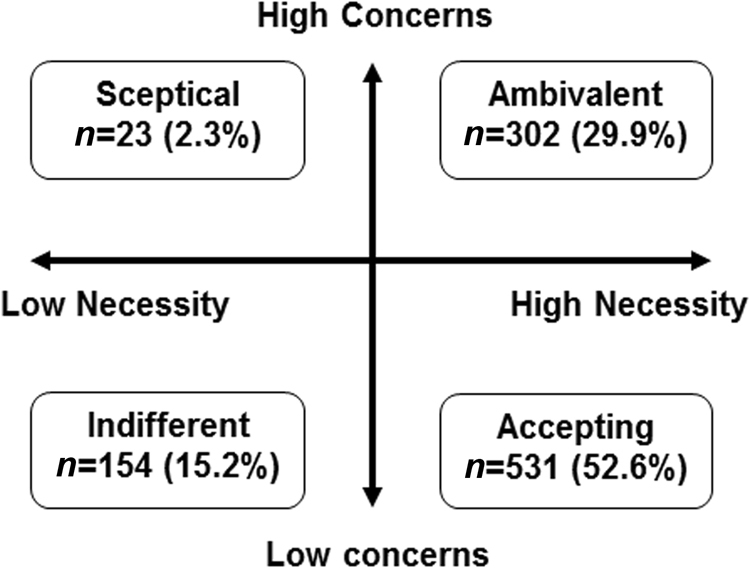
BMQ attitudinal analysis. BMQ, Beliefs about Medicines Questionnaire

Participants’ concerns and beliefs about the necessity of their asthma medication were collected using the necessity and concerns subscales of the BMQ. The overall median score was 3.60 for BMQ Necessity (IQR 3.00–4.00) and 2.67 for BMQ Concerns (IQR 2.00–3.22) (Table 2). The majority of participants (82.5%, *n = *833) were broadly convinced of the necessity of maintenance treatment, with only 17.5% (*n = *177) expressing strong doubts about personal need (BMQ Necessity scores; dichotomised at the midpoint). However, almost a third of participants had strong concerns about their current treatment (32.2% [*n* = 3 25] with high BMQ Concerns scores; dichotomised at the midpoint). When the BMQ Necessity and Concerns scores were combined in an attitudinal analysis, just over half of the participants were classed as ‘accepting’ of their condition (52.6%, *n = *531), approximately a third of participants were ‘ambivalent’ (29.9%, *n = *302) and fewer participants were ‘indifferent’ or ‘sceptical’ (15.2%, *n = *154, and 2.3%, *n = *23, respectively) (Fig. 1).

The majority of participants (73.5%, *n = *742) expressed a preference for a ‘once-daily medication that works as well as my current medication’ rather than a ‘twice-daily medication that works slightly better than my current medication’. The opposite preference, favouring the better twice-daily medication, was expressed by 26.5% of participants (*n = *268). The reasons behind these preferences were not elicited directly, but factors associated with preferences for once-daily or twice-daily medications were further explored, as described below.

### Significant univariate associations

Associations between participant scores are shown in Tables 3 and 4, and select associations are detailed below.

**Table 3:** Continuous variables significantly correlated with asthma control, treatment adherence and perceptual barriers to treatment, and associated with preference for once-daily asthma medication

Correlation between: Significance level *N* = 1010	ACT (asthma control)	ATIQ (treatment intrusiveness)	BMQ necessity (treatment necessity)	BMQ concerns (treatment concerns)	BMQ NCD (treatment evaluation)	MARS (adherence)	Preference for once-daily asthma medication
Asthma control (ACT score)		*r* = −0.369^a^***	*r* = −0.234^a^***	*r* = −0.343^a^***	*r* = 0.130^a^***	NS	NS
Adherence (MARS score)	*r* = 0.262^a^***	*r* = −0.422^a^***	NS	*r* = −0.506^a^***	*r* = 0.437^a^***		*t* = 2.970**
Age	NS	*r* = −0.177***	*r* = 0.126***	*r* = −0.125***	NS	*r* = 0.215^a^***	NS
Duration of asthma	*r* = 0.068^a^*	*r *= −0.111***	*r* = 0.075*	*r* = −0.0112***	NS	NS	NS
Number of lifetime asthma attacks	*r* = −0.272^a^***	*r* = 0.206***	*r* = 0.227***	*r* = 0.160***	*r* = 0.200^a^***	*r* = −0.161^a^***	NS
Number of asthma medications	*r *= −0.223^a^***	*r *= 0.141***	*r* = 0.129***	*r* = 0.121***	NS	*r* = −0.130^a^***	*t* = 2.418*
Number of HCP consultations^a^	*r* = −0.381^a^***	NS	NS	NS	NS	*r* = −0.075^a^*	NS

*ACT* Asthma Control Test™ (high score = good asthma control), *ATIQ* Asthma Treatment Intrusiveness Questionnaire (high score = high perceived treatment intrusiveness), *BMQ* Beliefs about Medicines Questionnaire (high BMQ necessity score = high perceived treatment necessity; high BMQ Concerns score = high level of concerns about treatment), *HCP* healthcare professional, *MARS* Medication Adherence Report Scale (high score = good adherence); *NCD* necessity-concerns differential, *NS* not significant, *r* Pearson's correlation coefficient (negative correlations indicate an inverse relationship), *t* test statistic

*P < 0.05

**P < 0.01

***P < 0.001

^a^ *n* = 1009

^b^ In the prior year

**Table 4:** Bivariate relationships between demographic and clinical variables and asthma control, treatment adherence, perceptual barriers to treatment and treatment preference. (a) Relationships between demographic variables and asthma control, treatment adherence, perceptual barriers to treatment and treatment preference; (b) Relationships between clinical variables and asthma control, treatment adherence, perceptual barriers to treatment and treatment preference

	(a)							
Comparison [degrees of freedom]: Significance level	*N* = 1010	Higher ACT score (better asthma control)	Lower ATIQ score (lower treatment intrusiveness)	Higher BMQ Necessity score (higher treatment necessity)	Lower BMQ Concerns score (fewer treatment concerns)	Lower BMQ NCD score (more negative treatment evaluation)	Lower MARS score (lower adherence)	Preference for twice-daily asthma medication
Males (vs. females)		NS	MWU = 158938.5***	NS	MWU = 151661.0***	NS	MWU = 03758.0***	NS
Country^a^	All countries	*F* = 4.70 [4]^b^ ***	*χ* ^2^ = 34.871 [4]***	NS	*χ* ^2^ = 42.449 [4]***	*χ* ^2^ = 38.814 [4]***	*χ* ^2^ = 48.643 [4]***	*χ* ^2^ = 38.84 [4]***
	Significant pairwise comparisons	Germany (vs. France/Italy)	Germany/UK (vs. Italy/Spain); France (vs. Italy)		France/Germany/ UK (vs. Italy/Spain)	Italy/Spain (vs. France/ Germany/UK)	Italy/Spain (vs. Germany)	UK (vs. Germany)
Cohabiting (vs. living alone)^c^		NS	MWU = 101935.0**	NS	NS	MWU = 125149.5*	NS	NS
Residents in rural areas (vs. urban areas)		*F*=3.95 [1]^d^*	MWU = 117460.5***	NS	MWU = 107909.5***	NS	NS	NS
Not employed full-time (vs. employed full-time)		NS	MWU = 140320.0**	NS	MWU = 137733.5*	NS	*χ* ^2^ = 27.873 [11]*	*χ* ^2^ = 6.90 [1]**
Smokers (vs. not current smokers)		NS	MWU = 116190.5**	NS	MWU = 113952.0*	NS	NS	NS

**(b**)								
Comparison [degrees of freedom]: Significance level	*N* = 1010	Higher ACT score (better asthma control)	Lower ATIQ score (lower treatment intrusiveness)	Higher BMQ Necessity score (higher treatment necessity)	Lower BMQ Concerns score (fewer treatment concerns)	Higher BMQ NCD score (positive treatment evaluation)	Higher MARS score (better adherence)	Preference for once-daily asthma medication
Asthma severity^e^	All severities	*F* = 41.17 [3]^f^***	*χ* ^2^ = 25.165 [2]***	*χ* ^2^ = 48.731 [2]***	*χ* ^2^ = 12.278 [2]**	*χ* ^2^ = 18.163 [2]^***^	*χ* ^2^ = 26.417 [3]^***^	NS
	Significant pairwise comparisons	Mild asthma (vs. moderate/ severe/unknown asthma severity)	Mild asthma (vs. moderate/ severe asthma)	Severe asthma (vs. mild/ moderate asthma)	Mild asthma (vs. moderate asthma)	Severe asthma (vs. mild/moderate asthma)	Unknown asthma severity (vs. known asthma severity)	
Medication regimen step^g^:	All steps	*F* = 5.26 [4]^b^***	*χ* ^2^ = 31.013 [4]***	*χ* ^2^ = 19.125 [4]**	*χ* ^2^ = 18.184 [4]**	NS	*F* = 7.701 [4]^b^***	NS
	Significant pairwise comparisons	Steps 2/3 (vs. Steps 4/5)	Steps 2/3 (vs. Step 5); Step 3 (vs. Step 1)	Step 2 (vs. Steps 3/4)	Steps 2/3 (vs. Step 5)		Steps 2/3 (vs. Step 5)	
Preference for twice-daily asthma medication (vs. once-daily medication preference)		NS	*t* = 2.689**	*t* = 4.581***	*t* = 4.372***	*t* = 6.886***	*t* = 2.970**	

*ATIQ* Asthma Treatment Intrusiveness Questionnaire, *BMQ* Beliefs about Medicines Questionnaire, *F* F-statistic, determined by one-way ANOVA, *MARS* Medication Adherence Report Scale (high score = good adherence), *MWU* Mann–Whitney *U* test, *NCD* necessity–concerns differential, *NS* not significant, *t* test statistic, *χ* ^2^ determined by Kruskal–Wallis H test#

*P < 0.05

**P < 0.01

***P < 0.001

^a^ Country of recruitment

^b^ *n* = 1005

^c^ Married/cohabiting**/**living with their partner (vs. single/widowed/divorced/separated)

^d^ *n* = 1008

^e^ “How has your doctor described your asthma?”

^f^ *n* = 1006

^g^ Stepwise treatment framework (GINA, 2015, summary of medication at each regimen step: step 1, SABAs alone or in combination with allergy treatment; step 2, ICS alone or leukotriene modifiers or ICS in combination with SABAs or allergy-induced asthma treatment; step 3, LABAs in combination with ICS or theophylline/related compounds or ICS in combination with allergy-induced asthma treatment; step 4, ICS in combination with LABAs and allergy-induced asthma treatment; step 5, omalizumab)

Asthma control correlated positively with reported adherence levels, duration of asthma and the number of required asthma medications, and correlated negatively with the numbers of lifetime severe asthma attacks and healthcare professional (HCP) consultations in the prior year (Table 3).

High levels of treatment adherence were positively correlated with age and negatively correlated with the numbers of severe lifetime asthma attacks, required asthma medications and HCP consultations in the prior year (Table 3). Participants preferring twice-daily asthma medication had higher treatment adherence levels than participants preferring once-daily medication (Table 4). Participants preferring once-daily medication were taking fewer asthma medications than those with a preference for twice-daily medication (test statistic = 2.418, *P = *0.016).

Perceived Treatment Intrusiveness levels correlated negatively with asthma control, duration and adherence, and correlated positively with the number of lifetime asthma attacks and required asthma medications (Table 3). Participants who were female, had mild asthma or preferred twice-daily asthma medication had lower ATIQ scores than participants who were male, had moderate/severe asthma or preferred once-daily asthma medication (Table 4).

Treatment Necessity scores were negatively correlated with asthma control and positively correlated with asthma duration, the number of lifetime asthma attacks and required asthma medications (Table 3). Participants who had severe asthma or preferred twice-daily asthma medication had higher levels of perceived treatment necessity than those with mild/moderate asthma or preferred once-daily asthma medication (Table 4).

Treatment Concerns scores correlated negatively with asthma control, adherence and asthma duration, and correlated positively with the numbers of lifetime asthma attacks and required asthma medications (Table 3). Participants who were female, with mild asthma, or preferred twice-daily asthma medication had reduced concerns about treatment vs. participants who were male, had moderate asthma or preferred once-daily asthma medication (Table 4).

The MARS adherence scores for participants who preferred once-daily and twice-daily asthma medication were 3.40 (IQR 2.90–4.00) and 3.60 (IQR 3.00–4.20), respectively. Participants preferring once-daily asthma medication had lower perceived treatment necessity, more concerns about treatment and higher perceived treatment intrusiveness than participants who preferred twice-daily asthma medication (Table 4).

### Structural equation modelling

Structural equation modelling (a statistical technique allowing multiple causal relationships to be specified simultaneously, for outcomes to act as both predictors and outcomes simultaneously and for measurement error to be included in the models)^31^ was used to test a theoretical, empirical model of associations between adherence, reported asthma control, healthcare seeking, preferences for once-daily vs. twice-daily maintenance asthma treatment, beliefs about inhaled corticosteroids and practical barriers to taking medication (asthma treatment intrusiveness). Two outlier cases that demonstrated very large deviations from multivariate normality (Mahalanobis distances >170) were removed. To produce the most parsimonious model, non-significant relationships between latent variables were systematically removed from the modelling output, and direct paths were added to improve the model fit. Two models best represented the data (Fig. 1; Supplementary Fig. S1), which are described below. All pathways in the final models were significant at *P < *0.01 after bootstrapping to adjust for bias arising from non-normal distributions.

The first model identified predictors of adherence, healthcare seeking and asthma control (Fig. 1a; Supplementary Fig. S1a). Goodness-of-fit statistics indicated that the data deviated significantly from model predictions (Supplementary Table S2), and the full model deviated significantly from a perfect fit (*χ* ^2^ = 15726.58 [degrees of freedom = 1243], *P < *0.001). Complex inter-relationships were indicated, which included an association between higher levels of concern about treatment and increased perceptions of treatment intrusiveness and reduced adherence, which in turn influenced asthma control. Perceived necessity of treatment, concerns about treatment and treatment intrusiveness influenced each other, adherence and asthma control. Asthma severity and adherence negatively impact on healthcare-seeking behaviour, while asthma control had a positive impact on healthcare-seeking behaviour. The proportion of variance in individual dependent variables indicated that the model predicted 36.1% of variance in self-reported adherence on the MARS scale, and 32.0% of variance in asthma control. However, only 4.4% of variance in healthcare-seeking was explained by the model.

**Fig. 2:**
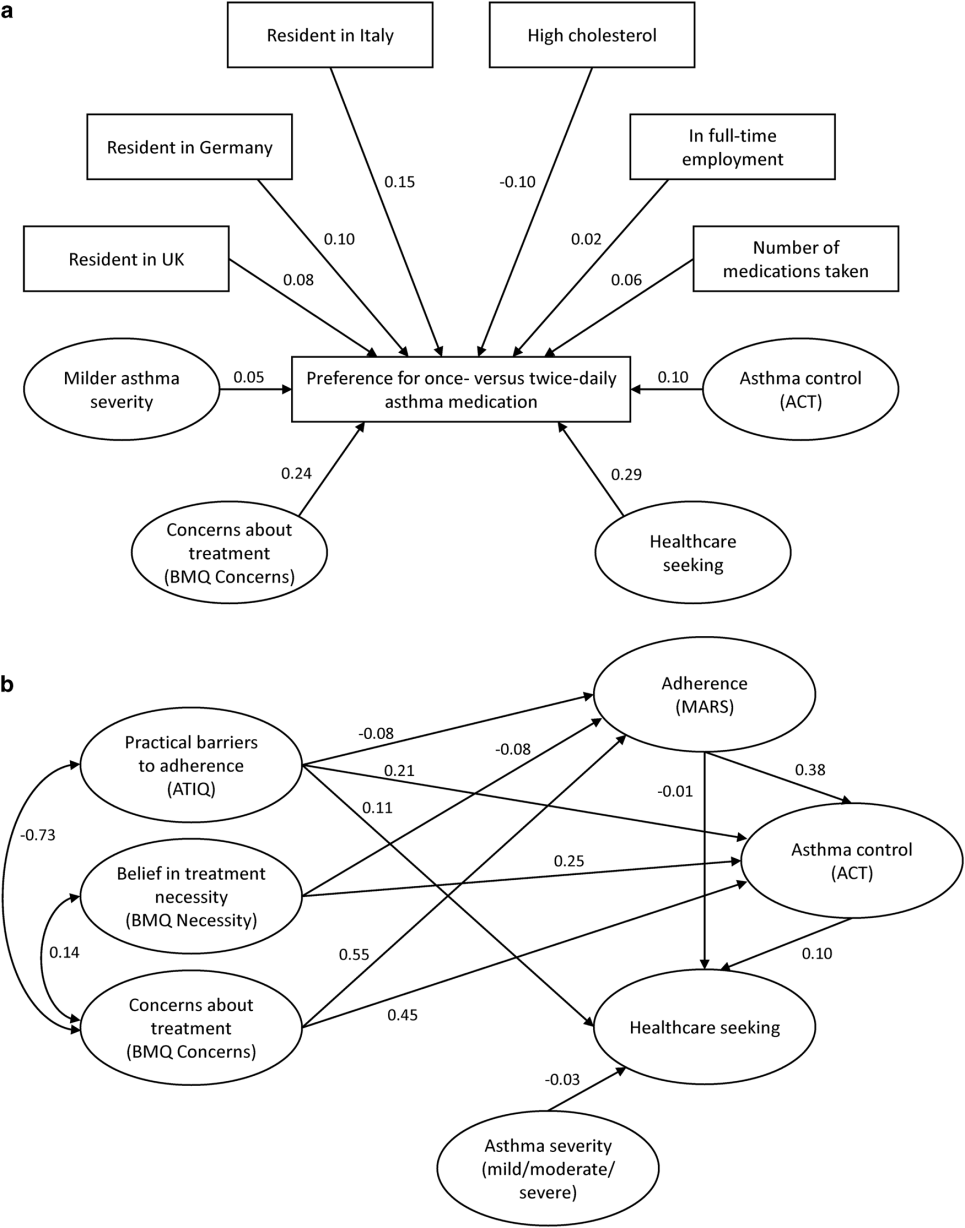
Simplified structural equation models identifying **a** predictors of adherence, healthcare seeking and asthma control, and **b** predictors of preference for once-daily vs. twice-daily treatment. ACT, Asthma Control Test™; ATIQ, Asthma Treatment Intrusiveness Questionnaire; BMQ, Beliefs about Medicines Questionnaire; MARS, Medication Adherence Report Scale. **a** Simplified structural equation model of association between adherence barriers, adherence, healthcare seeking, asthma control and asthma severity. All paths represent standardised regression weights of latent variables, corrected by bootstrapping, and are significant at *P = *0.01. Paths with a positive score have a positive impact of the connected variables, while negative scores indicate negative impacts. **b** Simplified structural equation model of predictors of preference for once-daily vs. twice-daily treatment. All paths represent standardised regression weights of latent variables, corrected by bootstrapping, and are significant at *P = *0.01. Positive paths are equivalent to an increased preference for twice-daily medication. Negative paths mean an increased preference for once-daily medication. ACT, Asthma Control Test™; ATIQ, Asthma Treatment Intrusiveness Questionnaire; BMQ, Beliefs about Medicines Questionnaire; MARS, Medication Adherence Report Scale

The second model identified predictors of preference for once-daily vs. twice-daily treatment (Fig. 1b; Supplementary Fig. S1b). Goodness-of-fit statistics indicated that the data deviated significantly from model predictions (Supplementary Table S3), and the full model deviated significantly from a perfect fit (*χ* ^2^ = 6029.50 [degrees of freedom = 420], *P < *0.001). Several factors were identified that influence preferences for once-daily or twice-daily medication: concerns about treatment, mild asthma severity, country of origin (UK, Italy or Germany), high cholesterol levels, full-time employment, higher number of medications taken, high ACT score and high levels of healthcare-seeking behaviour. The strongest predictors of preference for twice-daily asthma medication were concerns about treatment and healthcare-seeking behaviour. The included variables accounted for 21.2% of variance in preference for once-daily vs. twice-daily asthma medication. Participants who reported higher concerns and higher healthcare seeking tended to prefer twice-daily to once-daily asthma medication. (Fig. 2)

## Discussion

### Main findings

This post hoc analysis used survey data from people with asthma across five European countries to gain insight into adherence, asthma control, perceived treatment intrusiveness, healthcare-seeking behaviour and preferences for once-daily vs. twice-daily asthma medication. Although most participants reported good adherence to prescribed medication, the median MARS score of 3.40 (of a possible 5.00 for perfect adherence) suggested poor adherence by many patients; however, there was considerable variation in the data. The study identified variations in beliefs about treatment (necessity and concerns) and subjective treatment burden (Perceived Treatment Intrusiveness), and these perceptions were related to non-adherence as anticipated. The majority of participants (73.5%, *n = *742) expressed a preference for a ‘once-daily medication that works as well as my current medication’, compared with a ‘twice-daily medication that works slightly better than my current medication’.

Structural equation modelling identified complex inter-relationships between beliefs about medication, and treatment intrusiveness and adherence factors: positive beliefs about treatment (high necessity, low concerns) and higher adherence levels positively influenced asthma control, leading to less healthcare-seeking behaviour, whereas barriers to adherence and asthma severity had the opposite effect. In the first model, higher levels of concern about treatment were associated with increased perceptions of treatment intrusiveness and reduced adherence, which in turn influenced asthma control. The second model identified that higher levels of concern about treatment and healthcare-seeking behaviour were also predictive of preference for twice-daily asthma medication. The full models deviated significantly from perfect fit, as would be expected due to the difficulty of fitting complex models of human behaviours and beliefs precisely, and the high power to detect deviation from the model due to the large sample size. The model directly predicted approximately a third of the variance in adherence and asthma control, but only predicted a small proportion of the variance in healthcare seeking, indicating that further variables may be relevant for this. In general, structural equation modelling identified a wide variety of factors that were associated with a preference for once-daily asthma maintenance medication among participants.

### Interpretation of findings in relation to previously published work

The number of participants reporting a preference for once-daily treatment in this study was greater than that in other surveys of patients with asthma.^30^ We do not know the reason for this but it may be linked to the fact that, in this survey, the once-daily medication was described as being as effective as participants’ current medication. This description was not included in other surveys and may have led those participants to assume that a reduced frequency was associated with reduced efficacy of the medication. In our study, two key factors emerged as predictors of preference for more effective twice-daily asthma medication over once-daily asthma medication: high levels of treatment concerns and high levels of healthcare seeking. Participants who were more worried about their asthma medication, and were seeking more healthcare support, appeared to want more effective twice-daily asthma treatments in preference to once-daily dosing with a medication that would be as effective as their current treatment. Despite healthcare seeking being the strongest predictor of twice-daily asthma treatment preference in the structural equation model, there were no significant univariate associations between healthcare seeking and preference. The structural equation model may have been more able to detect an effect of healthcare seeking, whereas univariate analyses were performed for individual healthcare-seeking behaviours (including being hospitalised, calling an ambulance and staying overnight in hospital), which contributed to a single, composite latent variable in the structural equation model. Additionally, in the multivariate analysis, the effect of healthcare seeking may have become more important when other variables, which may interact or be confounded with healthcare seeking, were held constant.

In our study, relationships between asthma control, adherence to asthma medication, perceptual and practical barriers to adherence, and healthcare seeking largely conformed to predictions of the PAPA model and the NCF.^16^ Well-controlled asthma was associated with reduced healthcare seeking and increased adherence, compared with poorly controlled asthma, as previously reported.^32^ A separate study has previously found that, as expected, participants with a higher perceived need for their asthma maintenance treatment reported higher adherence to asthma medication, and participants who reported concerns about the potential adverse effects of their asthma medication or found their asthma medication intrusive were typically less adherent. Patients who perceive asthma to have potentially severe consequences and feel involved in their treatment decisions are known to be more likely to adhere to treatment.^33^ A recent study showed that negative beliefs about medication associated with non-adherence may not be addressed in asthma consultations with specialist nurses.^25^ Other studies have demonstrated interventions that appear to enhance necessity beliefs, reduce concerns and improve adherence.^34,35^

### Strengths and limitations of this study

The strengths of our analysis include the large number of participants (*N* = 1010), the collection of data in five European countries and the use of validated scales for adherence,^17,36^ asthma control,^37^ beliefs about medications^17^ and perceived treatment intrusiveness.^38^ A limitation of the study was that an existing cross-sectional observational study dataset was used (collected in 2011), which meant that causal and temporal relationships could not be ascertained. Also, the surveyed individuals may be unrepresentative of the whole population of patients with asthma as participants received financial incentives for completing the surveys, and information on socioeconomic status was not gathered. Other limitations were the use of self-reported illness and healthcare-seeking data, which may have been subject to reporting bias, and that the measures of preference used for once-daily vs. twice-daily treatment, and asthma severity, were single-item scales and so may not have produced consistent results.

### Implications for future research, policy and practice

Future studies should consider using multiple-item scales to determine asthma severity and to provide more sophisticated discrete-choice methodologies to better evaluate patients’ preferences. In addition, it may be possible to determine more specific reasons for non-adherence by measuring the forms of non-adherence (such as erratic non-adherence, intelligent non-adherence and unwitting non-adherence) and monitoring the efficiency of inhaler usage.^39^

Our findings are the first to include measures of practical barriers in the application of a PAPA model and indicated complex relationships between adherence and a number of factors. In particular, barriers to adherence (asthma treatment intrusiveness, concerns about asthma medication and doubts about the need for maintenance treatment) appear to have an important impact on adherence levels. Strong links between adherence and asthma control were identified, as was the role that asthma control, adherence to medication, asthma severity and asthma-treatment intrusiveness play in predicting healthcare seeking. Finally, various barriers to adherence, asthma control, demographic and clinical factors were associated with preferences for once-daily vs. twice-daily medication, with increased healthcare seeking and higher concerns about current treatment leading to a preference for a twice-daily medication that is more effective than current treatment. Future studies may use PAPA to assess barriers to adherence via the three phases of adherence: initiation, implementation and persistence.^6^ It would also be interesting to include exacerbation history in future studies, to identify whether frequent asthma exacerbations (which may be predicted by high blood eosinophil counts)^40^ are predictive of adherence or preferences for once-daily or twice-daily medication.

## Conclusions

Our findings confirm the utility of the Perceptions and Practicalities Approach (PAPA) and Necessity Concerns Framework (NCF) in explaining non-adherence linked to poor asthma control. To understand patient adherence to current medication and preferences for once-daily vs. twice-daily treatment, it is crucial to consider the perspectives of the individual patient. Supporting adherence to treatment requires a dual approach that considers perceptions (e.g., treatment necessity beliefs and concerns) as well as the practicalities (e.g., addressing the ability to adhere by making the regimen easy to use). This might be achieved through a three-step PAPA approach:^15^ (1) communicating a ‘common-sense’ rationale for why the treatment is necessary to achieve goals that are valued by the patient, (2) eliciting and addressing patient concerns, and (3) addressing practical barriers, for example, by simplifying the regimen and/or improving inhaler technique.

## Methods

### Study design

This post hoc analysis was conducted using cross-sectional, web-based survey data obtained in 2011, from five European countries (Germany, Spain, France, Italy and the UK). This market research survey did not require ethics committee or review board approval.

### Participants

Participants were recruited by a recruitment agency (Toluna Proprietary). The agency actively managed market research panels across all five target countries using advertisements in search engines, online banners, telephone recruitment and mail. The survey was administered online. Sampling was non-random, targeting a minimum of 200 respondents per country (100 male and 100 female); quotas were applied to ensure an approximately similarly sized sample across all five target countries and equal numbers of male and female respondents. Financial incentives were given for participation in the survey. The study methods were explained to all potential participants before obtaining informed consent. Participants were also informed that the anonymous data would be used in publications.

People aged between 18 and 55 years of age, with a self-reported asthma diagnosis (and able to define age at asthma diagnosis), who were taking at least one daily asthma maintenance treatment medication (zafirlukast, ciclesonide, beclometasone, budesonide, ipratropium bromide, fluticasone, formoterol plus beclometasone, sodium cromoglicate, salmeterol plus fluticasone, montelukast, formoterol plus budesonide, nedocromil sodium, omalizumab or salbutamol plus ipratropium), and who expected to experience asthma symptoms on most days (or every day) if asthma medication was not taken every day, were eligible to take part in the survey. People were excluded from the study if they self-reported a diagnosis of chronic obstructive pulmonary disease (COPD), if they were aged over 55 years (to exclude patients with COPD) and once gender/nationality/age quotas had been met.

### Measures

Participants were categorised in terms of the highest ‘Step’ of medication they were taking, based on current global guidelines.^5^

Medication adherence was measured using the MARS (^©^Professor Rob Horne; 10-item version; Supplementary Table S4).^36,41^

Self-reported preference for once-daily vs. twice-daily asthma maintenance (preventer) medication taking was elicited by asking participants the following: ‘If your doctor gave you a choice of two possible new preventer medications, which one would you choose?’. The two possible response options were as follows: ‘once-daily medication that works as well as my current medication’ and ‘twice-daily medication that works slightly better than my current medication’.

Self-reported asthma severity was elicited by asking participants whether their doctor had described their asthma as ‘mild’, ‘moderate’ or ‘severe’. Asthma control was measured by the ACT, as previously described.^37^ Self-reported healthcare seeking was elicited by asking participants how many times in the last year they had consulted HCPs regarding their asthma or needed to use emergency medical services due to their asthma (including being hospitalised, calling an ambulance, being sent to the hospital by their GP/asthma specialist doctor and staying overnight in hospital), and if they had, how many days their asthma had led them to be hospitalised in the last year.

To assess the extent to which taking asthma medication interferes with participants’ daily lives, practical barriers to medication taking were measured using ATIQ (^©^Professor Rob Horne), a tool which was adapted for use in asthma for this study (full details are provided in the Supplementary Materials).^38,42^ Participants were asked to indicate, on a five-point Likert-type scale (from ‘low’ = 1 to ‘high’ = 5), the degree to which each of 13 possible intrusions of asthma affected their daily lives. To obtain total scores, the items were summed; the potential range of scores was 13–65.

Participants’ perceived need for treatment was measured using the’Necessity’ subscale of the BMQ questionnaire,^17^ referring to participants’ preventer inhaler (Supplementary Table S4). Concerns about potential negative effects of treatment were measured using a modified ‘Concerns’ subscale of the BMQ questionnaire (^©^Professor Rob Horne),^17^ which included additional items resulting in a 9-item scale (Supplementary Table S4). Both scales were rated on 5-point Likert scales from ‘strongly agree’ to ‘strongly disagree’. For each scale, items were summed and then divided by the total number of items in the scale to obtain comparable scores (range 1–5), higher scores indicating stronger agreement with the scale construct.

All questionnaire scales had good reliability for all participants, regardless of location (Cronbach’s *α* scores were as follows: ATIQ = 0.954 [all countries *α* > 0.94]; MARS = 0.872 [all countries *α* > 0.850]; BMQ Concerns = 0.890 [all countries *α* > 0.86]; and BMQ Necessity = 0.811 [all countries *α* > 0.70]). The scales were dichotomised at their midpoint into high and low groups to describe the data, and then combined to form four attitudinal groups to describe the pattern of beliefs in the sample as established previously.^43^ A difference score (BMQ necessity–concerns differential; BMQ NCD) was also calculated to describe participants’ implicit evaluation of overall benefits vs. the risks of treatment.

### Analyses

Only complete surveys were analysed. All analyses were conducted using SPSS 22 (IBM, New York, USA) and the Amos™ structural equation modelling plug-in (Amos Development Corporation, Florida, USA), with *α* = 0.05 to test for statistical significance. To confirm internal reliability of the scales, Cronbach’s alpha values were calculated for BMQ Necessity, BMQ Concerns, ATIQ treatment intrusiveness, ACT and MARS. To enhance reliability of the BMQ scales, items were removed before calculating total scores, resulting in a 5-item necessity and 9-item concerns scale drawn from an initial pool of 23 potential items. Descriptive characteristics of the entire population on all key variables were summarised (including gender, age, nationality, employment, BMQ scores, ACT scores, ATIQ scores, MARS scores and healthcare-seeking reports). A NCD score, representing the overall evaluation by patients of their asthma medications, was calculated and used as a predictor in the univariate models. Univariate relationships were tested for significance using Pearson or Spearman statistics, *χ* ^2^ or F/*t*-tests or Mann–Whitney *U*-tests, dependent on distribution and variable type (categorical/continuous), between asthma control (as measured by ACT), reported adherence (as measured by MARS), preference for once-daily vs. twice-daily treatment, perceptual barriers (BMQ necessity and concerns scores) and practical barriers (ATIQ score), reported asthma severity, reported healthcare seeking and clinical and demographic factors. Levene’s tests for equality of variances were examined, and degrees of freedom were adjusted where variances were unequal between groups.

Structural equation modelling of multivariate relationships (including latent variables, error terms and analyses) was conducted. Maximum likelihood estimation was used to obtain estimates of model parameters. Bootstrapping using 500 samples from the original data set was used to adjust for non-normality in the data and to produce unbiased estimates of parameters and parameter errors. Mahalanobis distances were examined to identify outliers. Modification indices were used to identify potential adjustments to the model; only meaningful adjustments were considered (e.g., correlated errors for unrelated scales were not allowed). Non-significant relationships between latent variables were removed to produce the most parsimonious model (i.e., the model that included the fewest assumptions and variables while remaining able to explain the data). Goodness of fit indices (adjusted goodness of fit index, goodness of fit index, normed fit index, parsimony goodness of fit index, relative fit index and root mean square error of approximation) were used to ascertain the value of the final model. The consistency of the proposed causal structure in the two models was tested across country samples by conducting the separate structural equation models in each country and evaluating whether qualitative differences in the magnitude and direction of the relationships in the models exist. Models which failed to converge or resulted in positive definite matrices or squared multiple correlations greater than one (all indicative of specification errors) were excluded.

An exploratory model-building approach^44^ was used to check data were available for enough participants to ensure an adequate sample size for this post hoc analysis with the following assumptions: all of the determinants of adherence had only a small effect on this outcome when combined (*d* = 0.1) and six latent variables were being modelled (i.e., perceived treatment need, perceived treatment concerns, treatment intrusiveness, adherence, asthma control and healthcare seeking) using 30 observed variables (the items comprising the BMQ, ATIQ, MARS, ACT and healthcare-seeking scales). A minimum sample of 100 participants was needed to detect the model structure, and 526 participants were needed to detect the combined effect of the variables on adherence at *α* = 0.5 and power of 0.8.

### Data availability

Access to the data sets supporting the conclusions of this manuscript may be obtained via https://www.clinicalstudydatarequest.com/.

## Electronic supplementary material

Supplementary material
