# Supplementary material for: Modelling the effect of beliefs about asthma medication and treatment intrusiveness on adherence and preference for once-daily vs. twice-daily medication
Source: NPJ Prim Care Respir Med. 2017 Nov 14;27:61. doi: 10.1038/s41533-017-0061-7 (PMC5686129; doi:10.1038/s41533-017-0061-7)
Supplement: Supplementary file 1 — Supplementary material [file 41533_2017_61_MOESM1_ESM.docx]

**SUPPLEMENTARY INFORMATION**

**ATIQ – further information**

The Asthma Treatment Intrusiveness Questionnaire (ATIQ) scale was adapted from a scale originally developed by Professor Horne to assess patients’ perceptions of the intrusiveness of antiretroviral therapies (HAART; the HAART intrusiveness scale).^1^ This scale assesses convenience and the degree to which the regimen is perceived by the patient to interfere with daily living, social life, etc. The HAART intrusiveness scale has been applied to study differential effects of once- vs. twice-daily antiretroviral regimens^2^ and might be usefully applied to identify patients who are most likely to benefit from once-daily treatments.

References:

1. Newell, A., Mendes da Costa, S. & Horne, R. Assessing the psychological and therapy-related barriers to optimal adherence: an observational study. Presented at the Sixth International congress on Drug Therapy in HIV Infection, Glasgow, UK (2002).
2. Cooper, V., Horne, R., Gellaitry, G., Vrijens, B., Lange, A. C., Fisher, M. *et al.* The impact of once-nightly versus twice-daily dosing and baseline beliefs about HAART on adherence to efavirenz-based HAART over 48 weeks: the NOCTE study. *J Acquir Immune Defic Syndr* **53**, 369–377 (2010).

**Supplementary Table S1.** Asthma medications, reported by participants at the time of survey

| *Asthma medication* | n *(%)* |
| --- | --- |
| Salbutamol | 406 (40.2) |
| Beclometasone | 212 (21.0) |
| Salmeterol plus fluticasone | 209 (20.7) |
| Salbutamol plus ipratropium | 169 (16.7) |
| Formoterol plus budesonide | 166 (16.4) |
| Fluticasone | 135 (13.4) |
| Budesonide | 131 (13.0) |
| Montelukast | 123 (12.2) |
| Terbutaline | 107 (10.6) |
| Formoterol plus beclometasone | 96 (9.5) |
| Ipratroprium bromide | 83 (8.2) |
| Salmeterol | 64 (6.3) |
| Sodium cromoglycate | 58 (5.7) |
| Ciclesonide | 52 (5.0) |
| Zafirlukast | 44 (4.4) |
| Omalizumab | 43 (4.3) |
| Formoterol | 41 (4.1) |
| Beclometasone plus salbutamol | 37 (3.7) |
| Theophylline | 36 (3.6) |
| Nedocromil sodium | 32 (3.2) |
| Fenoterol plus ipratropium bromide | 27 (2.7) |
| Ketotifen | 19 (1.9) |
| Fenspiride | 19 (1.9) |
| Doxofylline | 19 (1.9) |
| Mepiphylline | 18 (1.8) |
| Flunisolide | 14 (1.4) |
| Cromoglicic acid | 10 (1.0) |
| Bombuterol | 4 (0.4) |
| Bamifylline | 3 (0.3) |
| Other | 35 (3.5) |

**Supplementary Table S2.** Goodness-of-fit statistics for structural equation model predicting adherence, healthcare seeking and asthma control

| *Goodness of fit statistic* | *Model value* | *Target value if fit perfect* | *Adequate fit* |
| --- | --- | --- | --- |
| GFI | 0.622 | 1.0 | >0.90 |
| AGFI | 0.597 | 1.0 | >0.90 |
| PGFI | 0.583 | 1.0 | >0.90 |
| NFI | 0.467 | 1.0 | >0.95 |
| RFI | 0.467 | 1.0 | >0.95 |
| RMSEA | 0.108 | 0.08 | 0.10 |
| Hoelter’s index | 85 | - | >200 |

Abbreviations: AGFI, adjusted goodness of fit index; GFI, goodness of fit index; NFI, normed fit index; PGFI, parsimony goodness of fit index; RFI, relative fit index; RMSEA, root mean square error of approximation.

**Supplementary Table S3.** Goodness-of-fit statistics for structural equation model predicting preference for once- vs. twice-daily asthma medication

| *Goodness of fit statistic* | *Model value* | *Target value if fit perfect* | *Adequate fit* |
| --- | --- | --- | --- |
| GFI | 0.769 | 1.0 | >0.90 |
| AGFI | 0.745 | 1.0 | >0.90 |
| PGFI | 0.695 | 1.0 | >0.90 |
| NFI | 0.388 | 1.0 | >0.95 |
| RFI | 0.366 | 1.0 | >0.95 |
| RMSEA | 0.115 | 0.08 | 0.10 |
| Hoelter’s index | 79 | - | >200 |

Abbreviations: AGFI, adjusted goodness of fit index; GFI, goodness of fit index; NFI, normed fit index; PGFI, parsimony goodness of fit index; RFI, relative fit index; RMSEA, root mean square error of approximation.

**Supplementary Table S4.** BMQ questionnaire subscales used to gather participants’ beliefs about medicines

| *MARS subscale* | | *BMQ Necessity subscale* | *Modified BMQ Concerns subscale* |
| --- | --- | --- | --- |
| Core scale | ‘I decide to miss out a dose’ | ‘My health, at present, depends on this inhaler’ | ‘I sometimes worry about the long-term effects of this inhaler’ |
|  | ‘I forget to take it’ | ‘My life would be impossible without this inhaler’ | ‘This inhaler gives me unpleasant side-effects’ |
|  | ‘I alter the dose’ | ‘Without this inhaler I would be very ill’ | ‘Having to use this inhaler worries me’ |
|  | ‘I stop taking it for a while’ | ‘My health in the future will depend on this inhaler’ | ‘This inhaler does more harm than good’ |
|  | ‘I take it less than instructed’ | ‘This inhaler protects me from becoming worse’ | ‘This inhaler is a mystery to me’ |
| Additional items^a^ | ‘I only use it as a reserve, if my other treatment doesn’t work’ |  | ‘People who are on preventer treatments should stop their treatment every now and again’ |
|  | ‘I only use it when I feel breathless’ |  |  |
|  | ‘I only use it when I need it’ |  | ‘This inhaler disrupts my life’ |
|  | ‘I try to avoid using it’ |  | ‘I can use as much of this inhaler as I need, without having to worry about becoming too dependent on it (Reverse)’ |
|  | ‘I use it before doing something which might make me breathless’ |  |  |

Responses were rated on 5-point Likert scales from ‘strongly agree’ to ‘strongly disagree’. ^a^Additional asthma-medication specific items.

Abbreviations: BMQ, Beliefs about Medicines Questionnaire; MARS, Medication Adherence Report Scale (high score = good adherence).

**Supplementary Fig. S1** Full structural equation models identifying (**a**) predictors of adherence, healthcare seeking and asthma control, and (**b**) predictors of preference for once- vs. twice-daily treatment

**a**
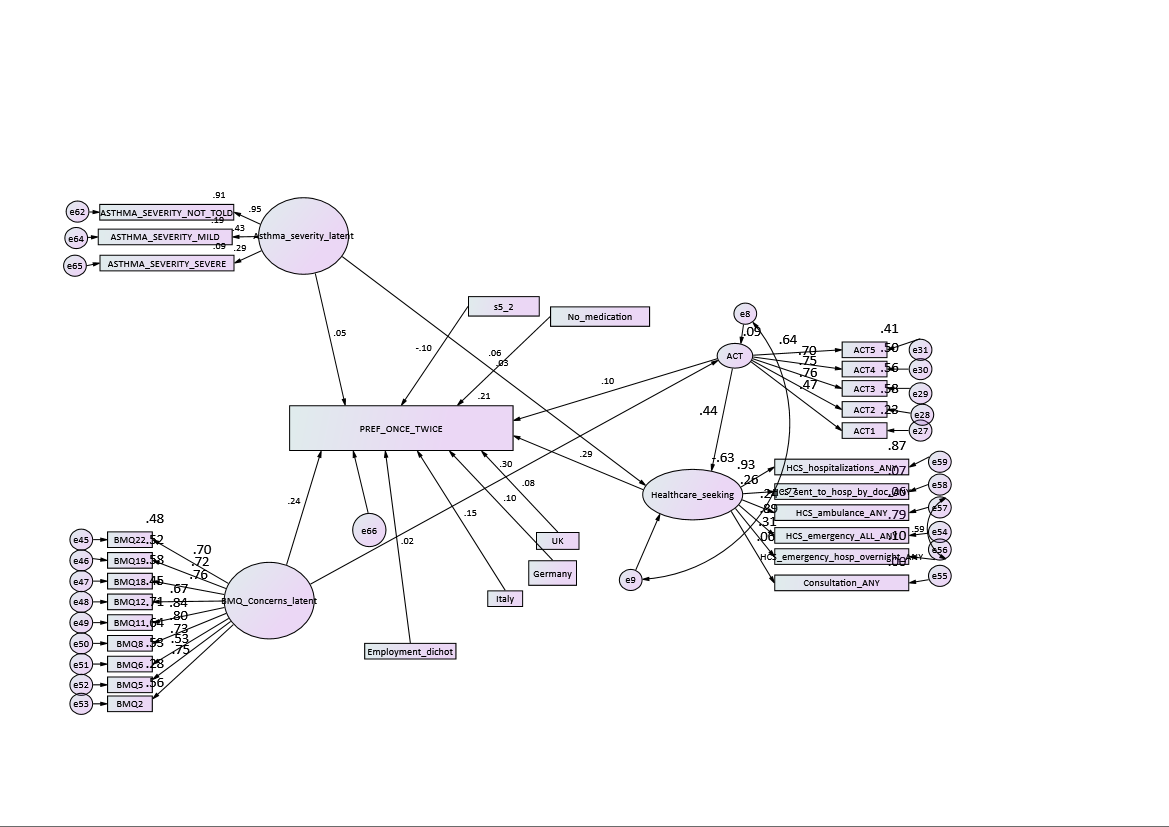


Full structural equation model of associations between adherence barriers, adherence, healthcare seeking, asthma control and asthma severity. All paths represent standardised regression weights of latent variables, corrected by bootstrapping, and are significant at *P* = 0.01.

**b**
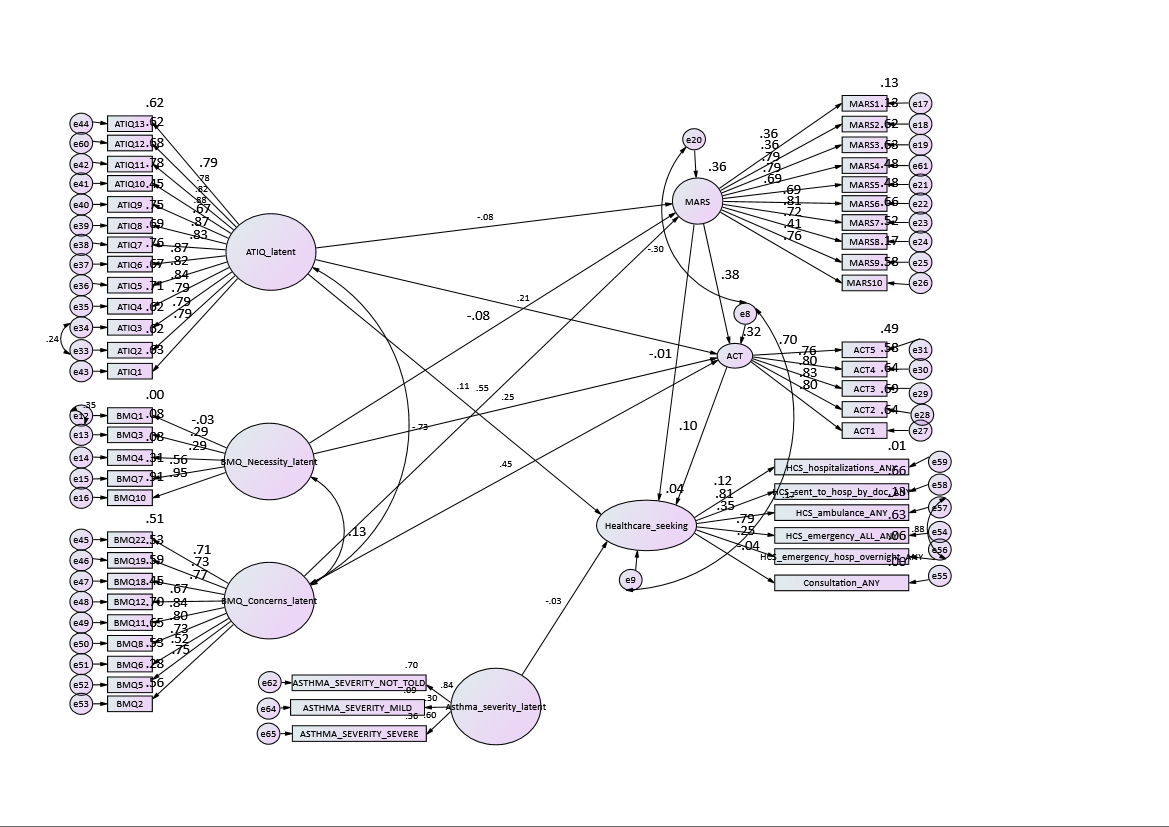


Full structural equation model of predictors of preference for once- vs. twice-daily treatment. All paths represent standardised regression weights corrected by bootstrapping and are significant at *P* = 0.01. Positive paths are equivalent to an increased preference for twice-daily medication. Negative paths mean an increased preference for once-daily medication. S5_2 = presence of high cholesterol.

ACT, Asthma Control Test™; ATIQ, Asthma Treatment Intrusiveness Questionnaire; BMQ, Beliefs about Medicines Questionnaire; HCS, healthcare seeking; MARS, Medication Adherence Report Scale
